# Second-Layer Palmar Graft for Fingertip Reconstruction: An Analysis of Aesthetic and Functional Outcomes

**DOI:** 10.7759/cureus.24257

**Published:** 2022-04-18

**Authors:** Subashini Rajendiran, Karthikeyan Gomathinayagam, Vishnubabu Gopalakrishnan

**Affiliations:** 1 Hand Surgery, Sri Ramachandra Institute of Higher Education and Research, Chennai, IND; 2 Hand and Reconstructive Surgery, Tamil Nadu Government Multi-Super-Speciality Hospital, Chennai, IND; 3 Plastic and Reconstructive Surgery, Sri Ramaswamy Memorial (SRM) Institutes for Medical Science (SIMS) Hospital, Chennai, IND

**Keywords:** keratin sparing split skin graft, glabrous dermal grafting, glabrous skin grafting, skin graft, second layer palmar graft, fingertip injuries

## Abstract

Background: Fingertip injuries are very common and require a stable and durable cover. The end result after reconstruction must be a painless finger with good aesthetic appearance. Skin loss in fingertip, with or without partial loss of pulp fat, is often managed with a split-thickness skin graft, which causes a poor aesthetic result due to color mismatch of the grafted skin in the glabrous volar skin of fingertip. The full-thickness and partial-thickness skin graft harvested from palmar skin provide color match but may cause donor site morbidity in the form of painful scar or contour deformity. Harvest of the second layer from the palm (intermediate part of dermis) allowed the first layer (epidermis with superficial part of dermis) to be reposed over the remaining dermis in palm, thus allowing closure of donor defect without any tension. This technique provides a good color match for the primary defect, along with reduced donor site morbidity.

Aim: The study aims to analyze the outcomes of second-layer palmar graft (SLPG) in patients with fingertip injuries.

Materials and methods: The retrospective study was conducted in January 2012 on 40 patients who underwent SLPG.

Result: The graft take was 100% in 36 patients with an average static two-point discrimination (2PD) of 6 mm. The average cosmetic visual analog score for the donor area was 100 and recipient site was 80.

Conclusion: The SLPG is a good surgical procedure for reconstructing fingertip defects, providing excellent aesthetic appearance and optimal function.

## Introduction

The reconstruction of fingertips depends on the size of the defect and the structures lost. Gonzalez et al. [1] stated that the goal of treatment of severely injured fingers is a reconstruction of the finger to normal appearance and function. The replacement of skin in the fingertip with a split-thickness skin graft harvested from non-glabrous (hair-bearing) skin is often complicated by the subsequent development of hyperkeratosis, hair growth, hyperpigmentation, hypertrophic marginal scarring, and, ultimately, breakdown of the graft [2]. Split-thickness glabrous skin grafts and, especially, thick split-thickness grafts result in significant donor site morbidity, along with prolonged healing, pain, hypertrophic scarring, and hyperpigmentation [3]. The full-thickness skin graft from the palm is limited in size [4]. The intermediate part of dermal grafting from the glabrous region (second-layer palmar graft), sparing the epidermal and superficial part of the dermis, has been advocated as a good alternative to avoid the problems of split-thickness skin grafting. A retrospective study was conducted in our institute to analyze the outcomes of second-layer palmar graft (SLPG) in cases with fingertip injuries.

## Materials and methods

The retrospective study was conducted in January 2012 at the Institute for Research and Rehabilitation of Hand and the Department of Plastic Surgery at Government Stanley Hospital, Chennai. The patients who underwent SLPG between July 2010 and June 2011 were recruited for the study. Patients who underwent other surgeries in addition to SLPG were excluded. A total of 49 patients were recruited, of which nine patients were lost for follow-up. The patients were operated on by multiple surgeons at different levels of expertise from consultants to first-year trainees. For this study, patient demographics, mode of injury, size of the defect, assessment of graft take, and duration of healing were recorded from the patients' records. Graft take was assessed by its adherence to the wound bed, and complete reepithelialization was considered as the endpoint of healing. At the time of final follow-up, graft site sensation was assessed by static two-point discrimination (2PD), and the patients’ scars at the donor site and the graft site were assessed using the cosmetic visual analog scale rated between 0 and 100 with 0 being the worst scar and 100 being the best scar [5]. Patients were also specifically questioned for donor site or graft site hyperesthesia. The follow-up period varied between six and 18 months.

Surgical technique

The surgery was done under axillary block or digital block with local anesthesia infiltration for the donor site. The anesthesia was decided after discussion with the patient and taking the patients' comfort into consideration. All patients underwent the procedure as outpatients, and none needed hospitalization.

**Figure 1:**
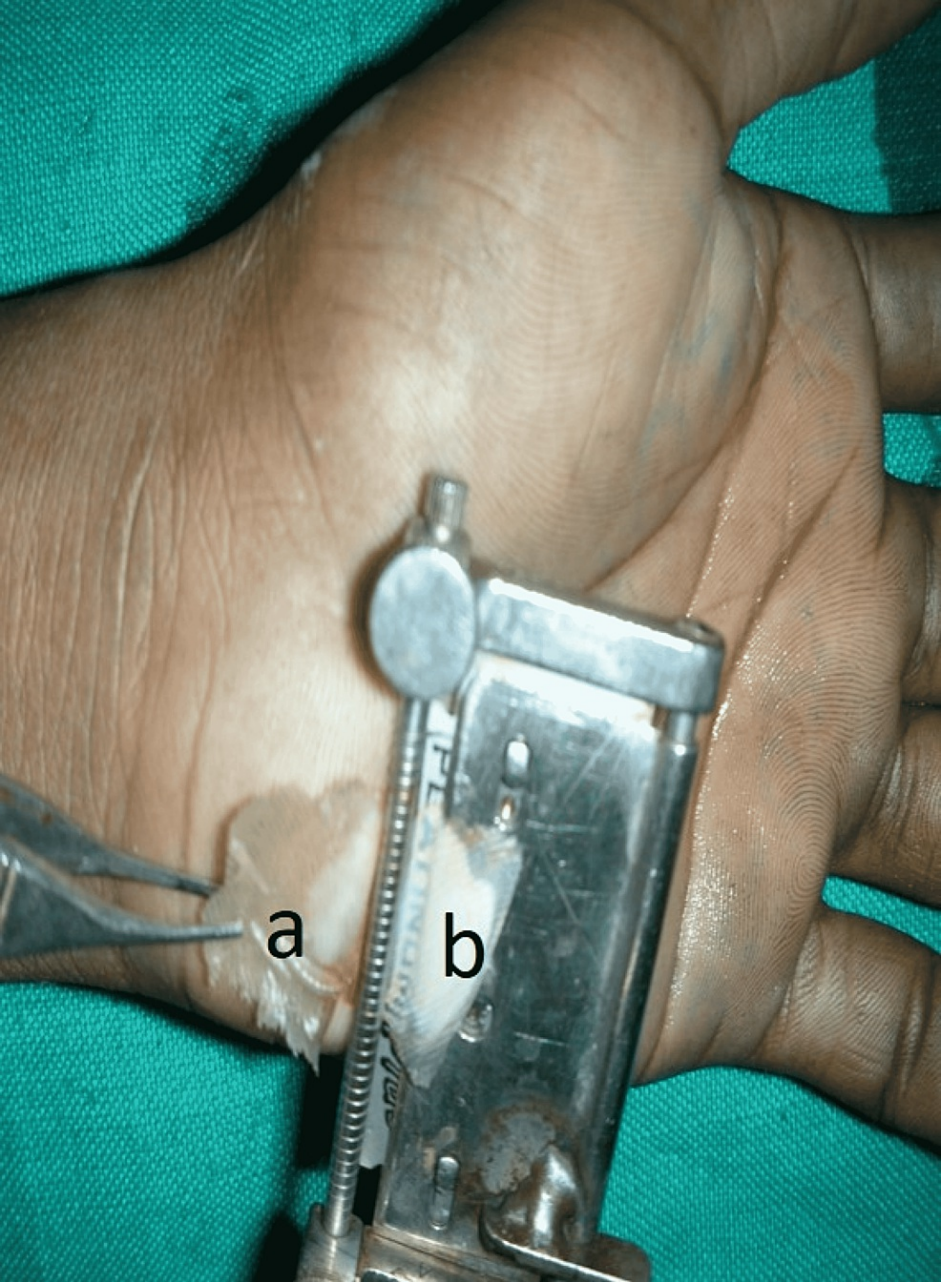
The harvested first layer (a) is held by toothed forceps. Harvest of second-layer palmar graft (b) at the donor site using manual dermatome.

The wound was debrided, and the injured finger was thoroughly washed. A template of the defect was taken. The first layer of palmar graft (epidermis with part of dermis) was raised from the hypothenar region by a manual dermatome and kept in continuity as a proximal-based flap (Figure 1). A template was placed and marking was done in the exposed dermis. Then the second layer of palmar graft (intermediate part of dermis) was harvested by the same manual dermatome, leaving behind a part of the dermis in the donor area, and applied over the defect.

**Figure 2:**
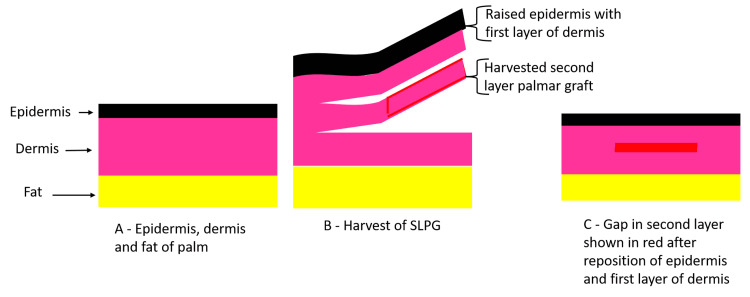
Diagrammatic representation of second-layer palmar graft harvest

Care was taken while placing the dermal graft so that the deeper part of the graft was in contact with the wound bed and the graft was not placed upside down. To avoid the upside-down placement of the graft, the harvested graft was placed facing upward in a saline gauze in a bowl. The graft was anchored with intermittent nonabsorbable sutures or tied-over dressing (Figure 2).

**Figure 3:**
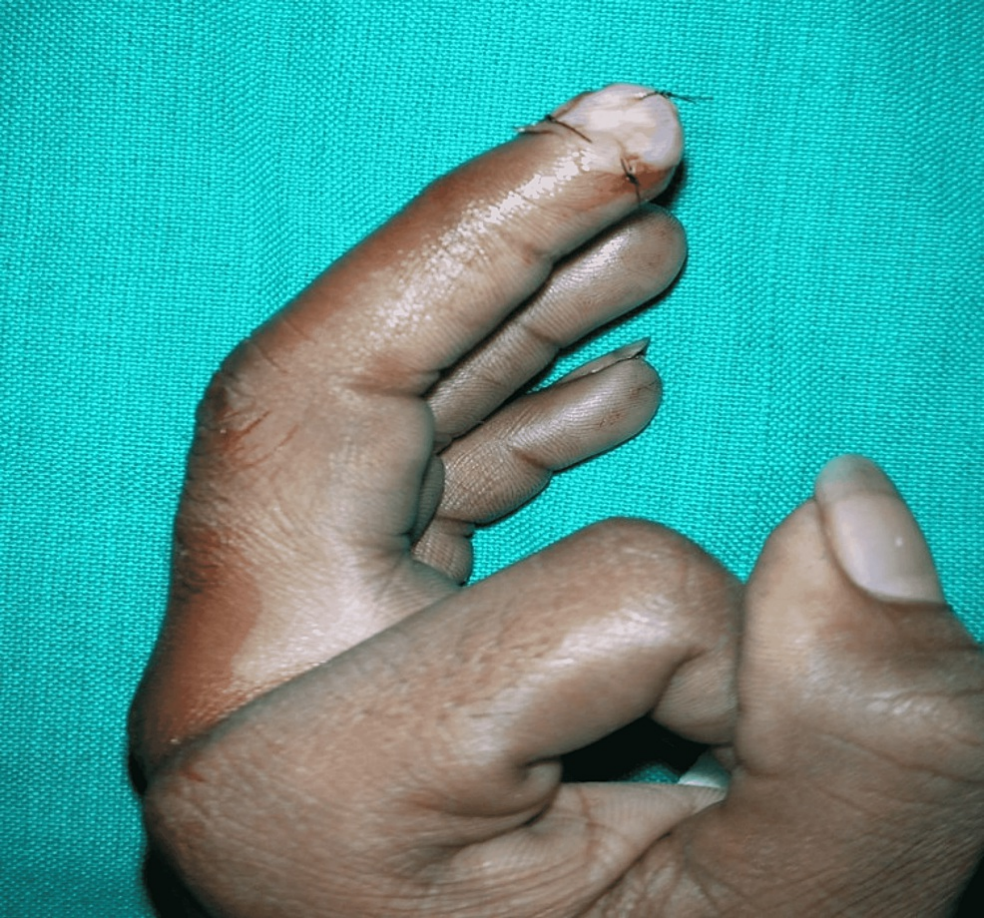
Anchorage of the second-layer palmar graft to the recipient site

The first layer of the graft was repositioned back and sutured (Figure 3). Paraffin gauze dressing was applied on the donor site and recipient site. The first dressing was done on the second or third post-operative day. Donor site and recipient site sutures were removed by 10-14 days postoperatively. Then, the patient was started on scar massage therapy.

## Results

**Figure 4:**
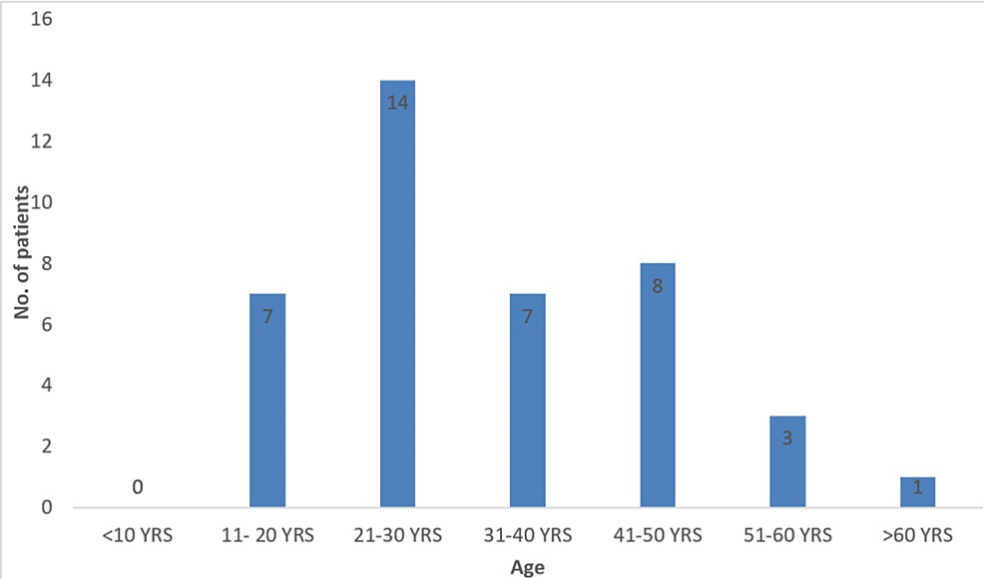
Analysis of age distribution of patients

Of the total 40 patients, 34 were males and six were females. Age group analysis showed that most of the patients were in the age group of 21-30 years (Figure 4). None of the patients had any associated comorbidities.

The mode of injury was work-place accidents for 32 patients and household accidents for eight patients. The time between injury and surgery was less than six hours in 32 patients, six to 12 hours in seven patients, and 12 to 24 hours in one patient. The injured finger was the little finger in five patients, the ring finger in nine patients, the middle finger in 10 patients, the index finger in nine patients, and the thumb in seven patients. The dominant hand of 26 patients (65%) was injured. The size of the defects varied from 1 cm x 1 cm to 2 cm x 2 cm, and they were confined to volar aspect of terminal phalanx region. There was an associated distal phalanx fracture in 15 patients (37.5%), but none of the defects had exposed bone.

**Figure 5:**
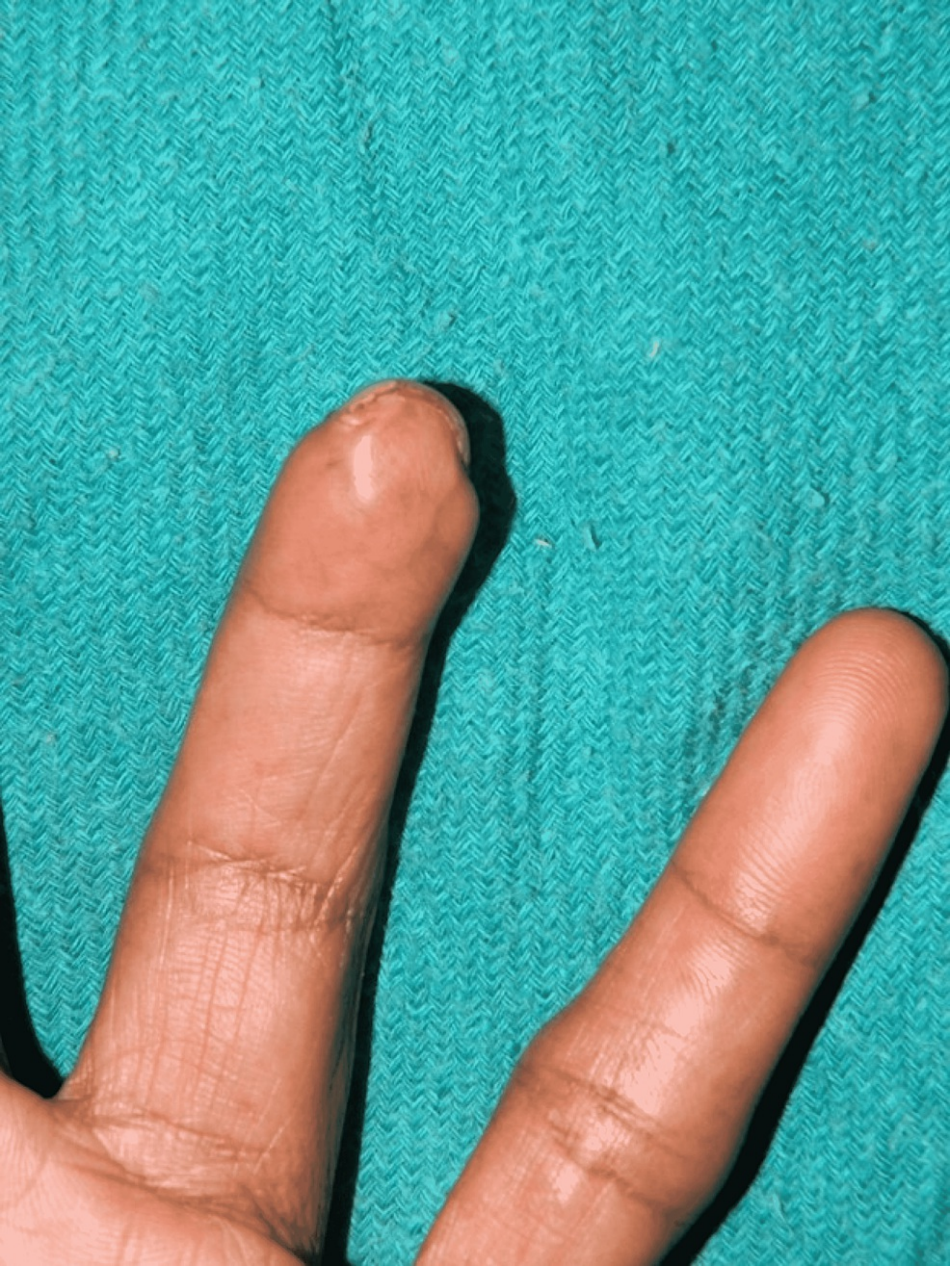
Recipient site after healing

**Figure 6:**
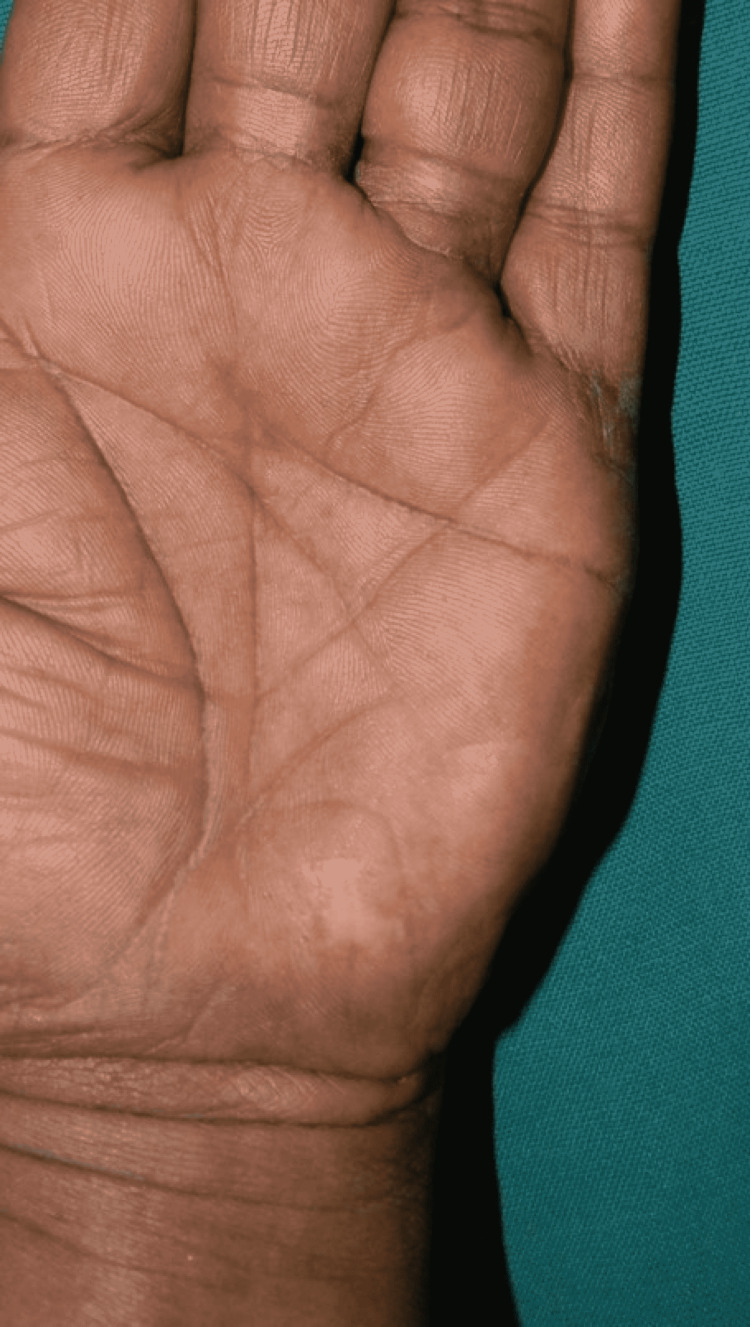
Donor site late follow-up

Of the 40 patients, 10 patients underwent surgery under axillary block, and 30 patients underwent surgery under digital block with local infiltration. The average duration of this surgery was 20 minutes (range: 15-30 minutes). There was partial graft loss in four patients, which healed with dressings. None of the patients needed secondary surgical procedures. The epithelialization time was between three and four weeks. None of the patients had problem with respect to donor site healing or donor site hypersensitivity. The average cosmetic visual analog score for the donor site was 100, whereas the average cosmetic visual analog score for the recipient site was 80. The late follow-up photos of recipient site and donor site are shown in Figures 5, 6, respectively. The average static 2PD was 6 mm (range: 4-12 mm).

## Discussion

The goal of treatment of any injured fingertip should be the restoration of a stable interface for object manipulation while looking as normal as possible [6]. When a wound is less than 1 cm^2^ without exposed barebone or tendon, healing by secondary intention is considered [6]. The other fingertip wounds require reconstruction in the form of split-thickness skin graft, full-thickness skin graft, composite grafts, dermal substitutes, or flaps.

In 1961, Moynihan [7] summarized that split-thickness skin graft was 80% successful by a long-term review of 100 fingertip injuries treated with split-thickness graft. In the current study, in the wound treated with second-layer palmar graft, partial graft loss was seen in only 10% of patients and the grafting was successful in 90% of cases. We do agree with Robotti et al. that a conspicuous black patch in the middle of a pale palm is often the single most frequently reported complaint by a trauma patient [2].

Patton (1969) advocated the reconstructive option of split-thickness skin graft from the hypothenar area based on their 20-year experience of usage of the procedure [8]. The donor site of the split-thickness skin graft can heal with hypertrophic and painful scars. Schenck et al. did an analysis of full-thickness skin graft from the hypothenar border and concluded that it provides a good aesthetic and functional result [9]. The limitation of full-thickness glabrous skin graft is the limited area available for harvest.

SLPG, described as glabrous dermal grafting by Wu et al. [3] and keratin sparing dorso-ulnar split skin graft by Goutos et al. [10], is a good alternative procedure with less donor site morbidity and good aesthetic outcome. We do agree with Martin-Playa et al. in avoiding the ulnar aspect of the hand as a donor site because it is often the surface on which the hand rests during activity [6].

The skin that lines the palm [11] of a human is typically 0.8-1.4 mm thick, while most of the skin in other parts of the body is only 0.1 mm thick. The glabrous skin differs from the skin on other parts of the body because it has a thicker dermis and its dermis is less elastic and more compact [3]. Wu et al. showed that all donor sites healed to completion without significant hypopigmentation, hyperpigmentation, or hypertrophic scarring [3]. Goutos et al. [10] showed that all donor areas healed within seven to 15 days, and only one patient developed donor site hyperesthesia. In the present study, all patients had good donor site healing, and none had hypersensitivity or hypertrophic scarring.

Schenck et al. [9] stated that 43% of cases had 2PD of 6 mm in their study where defects were resurfaced by full-thickness grafts from the ulnar border of the palm and concluded that the pattern of nerve plexus just below the basal layer of the epidermis is largely responsible for the extent and quality of its reinnervation. It is difficult to quantify the amount of this layer present in an SLPG. The average 2PD of 6 mm noted in the current study is comparable to 5.6 mm obtained in composite graft from the hypothenar region, 6 mm in cross-finger flap, and 5.7 mm in replanted fingers [12].

This study had a number of limitations. First, being a retrospective study, the selection bias for the patients undergoing the procedure could not be determined. Second, the sample size was small. A prospective randomized comparative study with split-thickness skin graft and other procedures can yield more meaningful conclusions.

## Conclusions

SLPG from the hypothenar region is a safe technique to resurface the fingertip injuries with good aesthetic outcomes, especially in terms of a color match. It has the advantage of donor site in the vicinity and primary closure of donor site defect. Also, the healing of donor and recipient sites is aesthetically and functionally good.
